# Drying kinetics and quality characteristics of ultrasound-assisted germination of quinoa grains: A study on functional properties, mineral content and bioactive compounds

**DOI:** 10.1016/j.ultsonch.2025.107393

**Published:** 2025-05-22

**Authors:** Jabir Khan, Yang Li, Palwasha Gul, Qingyun Li, Kunlun Liu

**Affiliations:** a Henan University of Technology, College of Food Science and Engineering, Zhengzhou 450001, P.R. China; b Mengniu Institute of Nutrition Science, Global R&D Innovation Center, Inner Mangolia Mengniu Dairy (Group) Co.Ltd., Beijing, 101107, P.R. China

**Keywords:** Quinoa, Pre-treatments assisted germination, Mathematical modelling, Minerals, Bioactive compounds, Functional properties

## Abstract

Quinoa (*Chenopodium quinoa Willd.*), an Andean grain, has garnered attention from cereal-based industries as a functional ingredient. This study evaluated functional properties, minerals, bioactive compounds and structural properties of Pre-treatments assisted germination of quinoa grains JQ-778 including, Only germinated seeds (OGS), soaked germinated seeds (SGS), ultrasound at 28 & 40 frequencies (US 28 kHz & US 40 kHz) for 30 min in a Biochemical-Incubator of 96-hour period at 25 °C, 12/12 h dark and light circle, followed by drying at temperatures 50 °C, 60 °C, 70 °C & combined (70, 60, 50 °C). Midilli-Kuck model shows the best fits on drying data closely followed by Hii model, respectively. According to the thermodynamic parameters entropy yielded negative values while Gibbs free energy & enthalpy decreased as the drying temperature increased. Ultrasonic pre-treatments significantly altered functional properties, minerals content and bioactive compounds of quinoa grains (p ≤ 0.05). In US group, quinoa’s magnesium, calcium, and potassium content increased from 124.17 to 143.59, 84.7–86.83, and 428.72–448.67 mg/100 g, respectively. Copper ranges from 0.77 to 1.04 mg/100 g, whereas iron 4.76–5.64, Manganese 3.09–3.51 and zinc 2.26–3.51 mg/100 g. Ultrasound assisted germination, especially US 28 kHz dried at 50 °C, showed the highest content of hydroxycinnamic acid, whereas US 40 kHz dried at 60 °C showed hydroxybenzoic acid of quinoa grains. US 40 kHz at 60 °C found rutin 22.62 µg/g, Quercetin 17.58 µg/g, while Kaempferol at 50 °C 24.51 µg/g, demonstrating a 2.5 to 4-fold increase in flavonoids and phenolic acid profiles in ultrasound pre-treated germinated quinoa grains. XRD showed no significant shifts in main peaks, although germinated samples showed intensity variations, suggesting crystallinity modifications. SEM pictures showed germinated quinoa aggregation at different temperatures, with a film-like substance covering and linking the aggregates, possibly a starch aggregates are coupled to lipids and incorporated in a protein matrix.

## Graphical abstract

Plan of the study, Biochemical Incubator (B.I); Light and dark circle (L.D); Only Germinated Seeds (OGS); Soaked Germinated Seeds (SGS); Ultrasound 28 kHz (US 28 kHz); Ultrasound 40 kHz (US 40 kHz).

**Figure:**
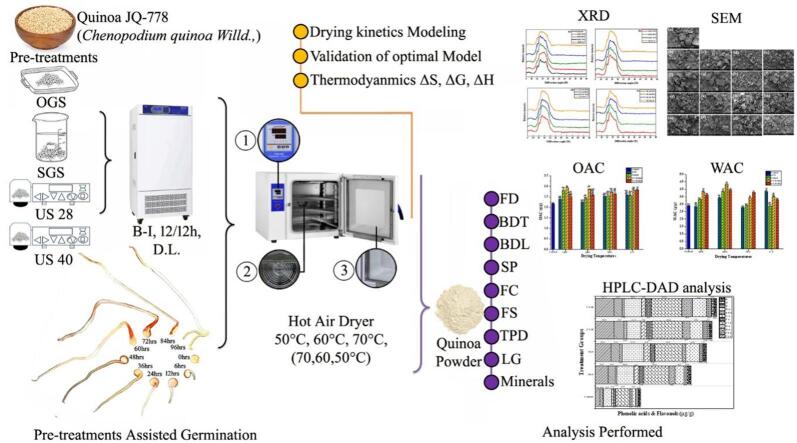

## 1 Introduction

Quinoa (*Chenopodium quinoa Willd.*) a well-known Andean grain, gained popularity due to high mineral content and phenolic content, has attracted worldwide attention in recent years [1,2]. Quinoa contains over 20-distinct phenolic compounds, most of which are phenolic acids and flavonoids like, quercetin & kaempferol [3]. Intake of quinoa grains play significant role in reduction risk of several diseases; which are mainly attributed to bioactive compounds like polyphenols, flavonoids, and minerals, have been highlighted in recent studies [4,5]. These advantages have led to the inclusion of quinoa into gluten-free food formulations [6,7]. In addition to its health benefits, quinoa has great promise as a functional component in a variety of products [3].

Nowadays, there is an increasing interest in making use of crops germination like quinoa to enhance their quality profile. Germination, a biological process known to improve the quality profile of quinoa, enhances the phenolic acids, flavonoids, and minerals [8,9]. A new trend in food processing is pre-germination treatments to improve the profile of germinated pseudo-cereal grains [10]. These pre-treatments and proper germination conditions improve quinoa grain quality profile [11]. In this context, food processing procedures such as SGS and ultrasound pre-treatments followed by germination had a substantial effect on grains composition [12]. However, each pre-treatment may affect grain quality differently [11]. A novel approach, ultrasound pre-treatment, improves seed nutritional profile as well as bioactive compounds accumulation [13,14]. Ultrasound, an effective, small energy consumption, sustainable and eco-friendly technology, has displayed considerable potentials on activating biosynthesis of nutraceuticals in seeds germination and improving nutritional profiles of germinated seeds [15]. Ultrasound pre-germination treatments have been currently investigated for mung bean & wheat [13,16]. Overall, the germination or sprouting process may be considered an effective strategy for enhancing the quality profile of grains [8,9,11].

In modern era, due to demand of high-quality, fast-dried food products, hot air, sun, microwave, and infrared drying are used to preserve food after germination, among which Hot air drying is a cost-effective [17,18]. To address the limits of standard drying methods for pre-treatments assisted germinated grains, hot air-drying technology is often used to improve the quality of food products and reduce drying time [19]. For example, Ultrasonic waves, a novel pre-treatment, include mass transfer during air drying. This technique involves soaking samples in hypertonic water or water before germination and drying, ultrasonic waves may excite medium particles and cause ultrasonic cavitation, which increases particle motion, changes material structure, and improves water diffusion. Power ultrasound's impacts on drying kinetics are mostly mechanical rather than thermal [20]. This system uses less thermal energy to improve moisture removal, preserving product quality.

Our previous study Khan et al. [21], investigated the effects of pre-treatments on the drying kinetics of quinoa grains, examined nutritional composition, colour attributes, antioxidant and enzymatic activities, as well as anti-nutritional and structural properties. Ultrasonic technology largely improves food drying and quality profile. Its application is still relatively rare. Grains quality must be studied in order to verify whether the treatment has affected properties of grains. Notably, the impact of ultrasound at different frequencies (US 28 kHz & US 40 kHz), followed by controlled germination (96 h under a 12/12 dark-light cycle) and drying temperatures and its impact on functional properties and bioactive compounds has not been investigated yet. Therefore, the present study first emphasizes identifying the most suitable drying kinetics model, then to study the impact of pre-treatments assisted controlled germination for (96 h under a 12/12 light–dark cycle) with focus on the functional properties, mineral content, and profiles of phenolic acids and flavonoids in quinoa grains.

## 2 Material and methods

### 2.1 Experimental conditions

Experimental conditions was based on our previous published study by Jabir khan et al. [21]. In short, quinoa seeds (JQ-778) were subjected to four pre-treatment conditions: OGS, SGS and ultrasonic pre-germination treatments US 28 kHz (Model: KMD-M3, Sn: 20220322036) and US 40 kHz (Model: KMD-M3, Sn: 20220322027) with 100 % amplitude for 30 min. This treatment protocol was chosen based on its previously demonstrated ability to enhance the quality of quinoa grains [21]. Quinoa grains were germinated at 25 °C in Biochemical Incubator (Model: SPX-250B-Z11) for 12/12 h dark and light circle for 96 h of germination. Subsequently, the germinated grains were dried in a hot air dryer (HAD) at temperatures of 50 °C, 60 °C, 70 °C, and combined temperatures (Graphical abstract). The drying process was monitored by recording the mass of the seeds hourly until achieved constant weight of each sample. Each experiment was conducted in triplicate to ensure reproducibility and statistical reliability.

A core aspect of this study was, application of 15 drying models to describe the moisture loss over time, compared to the 7 models used in the previous study. The models that best fit the experimental data were selected based on highest R^2^ values (greater than 0.99) with lowest X^2^, RMSE & RSS. Table 1 shows the applied, among them two were identified most effective for drying kinetics of germinated quinoa seeds. This refined modelling approach allowed for a more accurate prediction of drying behaviour and provided valuable insights into optimizing the drying process, especially in the context of different pre-treatment methods and drying temperatures. Compared to the previous study, this approach offered a deeper understanding of moisture loss dynamics and contributed to the optimization of drying conditions for quinoa and other similar seeds.

**Table 1:** Applied thin layered models applied on dried quinoa grains.

S. No	Model	Models Formulas	References
1	Newton	MR = exp(−*k*t)	[23]
2	Logarithmic	MR = *a*exp (−*k*t) + c	[24]
3	Two-term exponential	MR = *a*exp(−*k_0_* t^n^)+(1-*a*)exp(−*k_1_*t^n^)	[25]
4	Page	Mr = exp (−*k*t^n^)	[26]
5	Henderson & Pabis	Mr = *a*exp (−*k*t^n^)	[27]
6	Modified Henderson and Pabis	MR = *a*exp(−*k*t) + *b*exp(−*g*t) + *c*exp (−*h*t)	[28]
7	Wang and singh	MR = 1 + *a*t + *b*t^2^	[29]
8	Silva	MR = exp(−*a*t-*b*$\sqrt{t}$)	[30]
9	Midilli-Kuck	MR = aexp(−*k*t) + *b*t	[31]
10	Aghbashlo	MR = exp(*k*_1_t/1 + *k*_2_t)	[32]
11	Hii	MR = *a*exp(−*k_1_*t^n^) + *b*exp(−*k_2_*t^n^)	[33]
12	Two term	MR = exp(−*k_1_*t) + *b*exp(−*k_2_*t)	[34]
13	Page Midilli	MR = *a*exp(−*k*t) + *b*	[35]
14	Approximation of diffusion	MR = *a*exp(−*k*t) + (1 − *a*)exp(−*kb*t)	[36]
15	Verma	MR = *a*exp(−*k*t) + (1 − *a*)exp(−*g*t)	[37]

Furthermore, this study also aimed to determine the thermodynamic properties of quinoa seed drying, including changes Gibbs free energy (ΔG), entropy (ΔS), and enthalpy (ΔH), changes by using (Eq. (1)–(3)) [22]. These thermodynamic parameters provide additional insights into the energy changes during the drying process of quinoa grains.

(1) $$\Delta G=R\times T\times In\left(\frac{k\times hp}{T\times kb}\right)$$

(2) $$\Delta S=\frac{\Delta H-\Delta G}{T}$$

(3) ΔH=Ea-RT

*Kb* & *hp* denote Boltzmann constant, 1.3806 × 10^–23^ J/K & Planck constant 0.6262 × 10^−34^ J/s.

### 2.2 Techno-functional properties

#### 2.2.1 Flour dispersibility

For flour dispersibility, the protocols of Sharma et al. [38] were used with slight modifications. In short, 30 mL of DW was poured to a stoppered measuring cylinder containing 1 g of the sample, and the mixture was violently mixed. Mixture was then let for 3hrs, and the volume was determined using (Eq. (4)).

(4) $$Flour\;dispersibility\;\%\;=\frac{30-volume\;of\;settled\;particle}{30}\times 100$$

#### 2.2.2 Bulk density & tapped

Bulk and tapped densities were calculated using Ajay et al [39], calculations with minor modifications. 2 g of each sample was freely poured into a 50 ml glass graduated cylinder to measure loose bulk density. The cylinder was tapped 5 times from 10 cm. Volume was recorded from the cylinder for calculating bulk density and expressed as g/cm^3^. Tapped density was obtained by manually tapping the cylinder 100 times from 10 cm away until constant volume was reached and expressed as g/cm^3^.

#### 2.2.3 Swelling power

Swelling power were examined by using protocols of Singh et al. [40], with slight modifications. In short, 2 g sample with 20 ml distilled water (DW) were mixed in a centrifuge tubes. After 30 min of incubation at 50 °C, the tube was then cool to ambient temperature. The mixture was then centrifuge at 3000 × g for 20 min, and data were calculated using (Eq. (5)) and expressed as g/g.

(5) $$Swelling\;Power\;=\frac{Weight\;of\;the\;paste}{Initial\;weight\;of\;the\;sample}$$

#### 2.2.4 Foaming capacity and foaming stability (FC & FS)

The technique described by Narayana et al. [41], was used to determine FC & FS. 50 ml of DW and 1 g of quinoa powder were placed in a graduated cylinder at 30 ± 2 °C. To produce foam, the suspension was shaken and stirred for 5 min. By measuring the volume of the foam after 30 s of whipping, the FC was calculated using (Eq. (6)): The mixture was let to sit for an hour in order to assess the FS using (Eq. (7)): Every analysis was carried out in duplicate.

(6) $$FC\left(\%\right)=\frac{Volume\;after\;whipping-Volume\;before\;whipping}{Volume\;before\;whipping}\times 100$$

(7) $$FS\left(\%\right)=\frac{Foam\;volume\;after\;1\;hour\;of\;whipping}{Initial\;foam\;volume}\times 100$$

#### 2.2.5 Particle true density

The water displacement method was used to figure out the actual density of quinoa by following [39]. All tested conditions samples of known weight were added to a 500 cm fractionally graduated measuring cylinders separately, which contained 250 cm of DW. The water rise indicated the quinoa powder actual volume, data was calculated using; Particle true density = Weight of the sample/Volume of the sample and expressed as g/cm^3^.

#### 2.2.6 Determination of least gelation capacity

The least gelation concentration was examined by using the protocols of Chinma et al. [42]. Sample suspensions of was prepared with different percentage (2, 4, 6, 8, 10, 12, 14, 16, 18, 20, & 22) (w/v) in 5 ml of DW. The test tubes containing the suspension were rapidly cooled under tap water after being heated for an hour in a boiling water bath followed by further cooling for 2 h at 4 °C. Least gelation concentration was determined if the sample from the inverted test tube did not slide or fall &data were expressed;  No gel;  Partial gel;  Strong gel.

#### 2.2.7 Water absorption capacity (WAC) & oil absorption capacity (OAC)

WAC and OAC was measured using protocols of Singh et al. [40], with minor modifications. In short 2 g of sample were mixed with 20 ml DW, vortexed for 1 min, then centrifuged at 5000 × g for 20 min. WAC was expressed as g/g of water absorbed per g of sample. OAC of quinoa was evaluated similarly to WAC, but sunflower oil was used instead of DW & was expressed as g/g.

### 2.3 Minerals

Mineral content as examined using the protocols of Tazrart et al. [43]. An atomic absorption spectrometer (A6800) was used to measured following mineral: Magnesium (Mg), Calcium (Ca), Iron (Fe), Copper (Cu), Potassium (K), Manganese (Mn), and Zinc (Zn). For this, 5 g of the sample was converted to ash in a microwave (G80F20CN2L-B8-R0) oven at 550^◦^C, 0.1 g of different tested condition samples was digested with nitric acid. Finally, micronutrients were identified by comparing their emission spectra to a standard curve.

### 2.4 HPLC-DAD analysis

The extraction procedure detailed by Speranza et al. [44], was used to determine phenolic acids and flavonoids profile. Filtered samples were analysed using a HPLC (Waters Alliance e2695) with a photodiode array detector, Quaternary Pump, degasser, auto sampler, and auto injector (E2695). A reversed phase C-18 column (4.6 × 3 250 mm, ZPC-C18 5 µm) was used for separation of phenolic compounds. A 1000 µg/mL standard solution of phenolic compounds were prepared in methanol. The working standard solutions were prepared by diluting the stock solution at 0.1, 2.5, 5, 50, 100 and 1000 g/ml. The black bottles held all conventional solutions at 4 °C. Mobile phase A (0.1 % formic acid) and B (100 % methanol). Gradient program was: 0.0–5.0 min, 0–10 % B; 5.0–20.0 min, 40 %B; 20.0–32.0 min, 45 %B; 32.0–45.0 min, 50 %B; 45.0–70.0 min, 80 %B; 70.0–75, 5 %B. First, methanol dissolved in the standards. The calibration curve was diluted in mobile phase. All samples were injected at 5 µl and flowed at 0.2 mL/min. All standards were run at 254, 280, 330 & 365 nm. The standards were selected according to the best performance on each wavelength. In short, the standards were detected by 254, 280 & 330 nm. In which 254 nm was selected according to the performance of standards and samples. In short, the standards and samples were detected by 254, 280 & 330 nm. Detection wavelength was selected according to the best performance of each sample and standards with the following Caffeic acid (254 nm, 31:56), Chlorogenic acid (330 nm, 28.14), Ferulic acid (254 nm, 39.41), Isoferulic acid (254 nm, 41.17), *p*-coumaric acid (330 nm, 47.33), Ellagic acid (254 nm, 14.32), Gallic acid (254 nm, 15.86), P-Hydroxybenzoic acid (280 nm, 36.02), protocatechuic (280 nm, 27.10), Kampferol (254 nm, 66.37), Quercetin (254 nm, 69.42), Rutin (254 nm, 64.46). All analysis was done in replicate. The quantification of identified peaks was carried out through area normalization and results were calculated in µg/g of flour dry basis.

### 2.5 Crystalline property XRD

The XRD experiments were carried out in a manner similar to the approach outlined by [45]. The crystalline structure of pre-treated assisted germinated quinoa powder was studied using X-ray diffraction (XRD) (Rigaku, Tokyo, Japan) at a target voltage of 40 kW, a target current of 100 mA, a scan range of 5–60 (2θ), and a scan rate of 2°/min from 5° to 40°.

### 2.6 SEM

All samples microstructure including (Control, OGS, SGS, US 28 & US 40) pre-treated before germinated dried at various temperatures was detected using a SEM (FEI, Quanta 250FEG) at the Henan University of Technology by following protocols of [21]. Quinoa grains powder was placed on a SEM stub using double-sided tape putter-coated with gold (Ted Pella 108auto) & studied at 3.00 kV with 2000× magnification & scale bar: 50 µm.

### 2.7 Statistical analysis

Analysis was done in replicate & data were expressed in means & standard deviations. One Way analysis of variance and a Tukey's test was use for analysis (p ≤ 0.05). Significant differences were denoted by distinct letters. All analyses & drawing were per formed by IBM SPSS Statistics Version 23.0 software and Origin 2021 software, respectively.

## 3 Results and discussions

### 3.1 Mathematical modelling

#### 3.1.1 Drying process behaviour

**Fig. 1:**
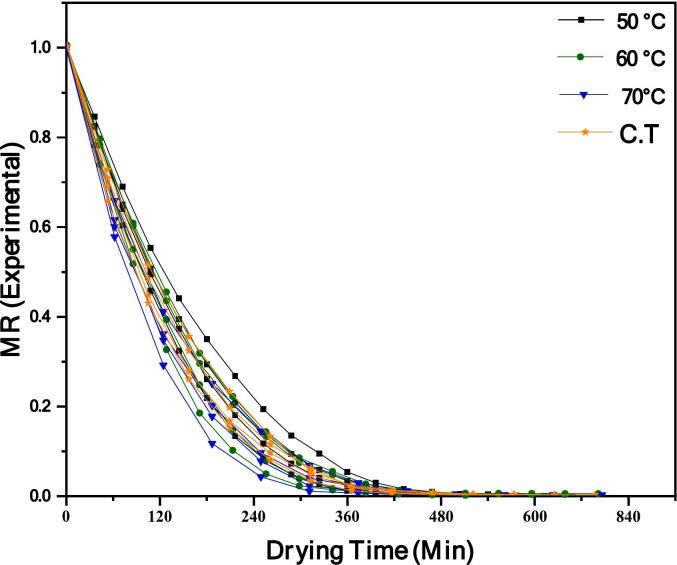
Moisture experimental vs drying time, OGS, SGS, US 28 kHz and US 40 kHz influenced by HAD at 50 °C, 60 °C, 70 °C & C.T.

Prior to approaching the study of drying of any food, its moisture content must be identified and assessed; as it mathematically represents the relationship among the activity of water and equilibrium moisture ratio [46]. Fig. 1 illustrates the drying curves at all drying temperatures. Which exhibit an exponential trend, drying time reduces as temperature increases to achieve equilibrium moisture content. In general, 50 °C takes twice as long as 70 °C to achieve the same moisture content. The drying rate curves steepened with temperature. Similarly, Carvalho et al. [14], also discovered that ultrasonic-assisted germinating barley grains followed by HAD significantly reduced drying time compared to other conditions. Applying ultrasonic waves to causes variations in oscillation speed, microfluidics, and pressure at the interface, resulting in mechanical agitation [47]. This agitation facilitates the transfer of water from the sample surface to the air, thus accelerating the drying process. Regarding US pre-treatments assisted germination followed by HAD enhanced the quality of grains and decrease the drying time, similar results has been observed for barely by [14], mung bean [13], and wheat [48].

#### 3.1.2 Best fitting Mathematical modelling

Mathematical models can help us predict the drying effect through statistical analysis. The drying kinetics of pre-treatments assisted germinated quinoa grains dried at all temperatures in hot-air drying were described using drying models. Model quality was assessed using R^2^, reduced X^2^, RMSE & RSS. High R^2^, low X^2^, RMSE, &BRSS suggest an excellent model fit for product drying kinetics. Even though all the selected models fitted satisfactorily with the experimental data. Statistical results of the two best-fitting models (Midilli-Kuck & Hii model), including model constants (a, b, n, k, k1, k2 & c) with R^2^, RMSE, X^2^ and RSS are shown in Table 2.

**Table 2:** Averages of the best-fitting models' statistical analysis data for quinoa dried at all temperatures in a HAD.

Models	Drying Temperatures											
	50 °C						60 °C					
	N/O	Constants & Coffi.	R^2^	X^2^	RMSE	RSS	N/O	Constants & Coffi.	R^2^	X^2^	RMSE	RSS
Midilli-Kucuk	T2	a = 0.34439 k = 0.56063 b = 0.51559 n = 0.03362	0.9996	0.3 × 10^−3^	0.001	0.6 × 10^−3^	T3	a = 0.34769 k = 0.67853 b = 0.61456 n = 0.03951	0.9995	0.9 × 10^−5^	0.001	0.6 × 10^−3^
	T6	a = 0.39317 k = 0.61135 b = 0.54874 n = 0.03464	0.9997	0.2 × 10^−4^	0.001	0.3 × 10^−3^	T7	a = 0.31638 k = 0.75598 b = 0.71969 n = 0.03756	0.9997	0.9 × 10^−5^	0.4 × 10^−3^	0.5 × 10^−5^
	T10	a = 0.36473 k = 0.65729 b = 0.56134 n = 0.03836	0.9995	0.001	0.001	0.6 × 10^−3^	T11	a = 0.35249 k = 0.69826 b = 0.55074 n = 0.04353	0.9997	0.001	0.1 × 10^−3^	0.001
	T14	a = 0.34381 k = 0.70228 b = 0.65191 n = 0.03748	0.9998	0.1 × 10^−4^	0.001	0.001	T15	a = 0.31877 b = 0.71958 n = 0.67906 n = 0.04265	0.9998	0.9 × 10^−5^	0.4 × 10^−3^	0.001
Hii Model	T2	a = 0.36581 k1 = 0.47256 n = 0.10967 k2 = 0.03230 c = 0.04938	0.9994	0.2 × 10^−3^	0.3 × 10^−3^	0.001	T3	a = 0.37295 k1 = 0.56973 n = 0.11620 k2 = 0.03675 c = 0.04020	0.9993	0.002	0.002	0.001
	T6	a = 0.40567 k1 = 0.51904 n = 0.11394 k2 = 0.03192 c = 0.04945	0.9994	0.2 × 10^−3^	0.001	0.001	T7	a = 0.33808 k1 = 0.66542 n = 0.10257 k2 = 0.03546 c = 0.04049	0.9993	0.8 × 10^−3^	0.001	0.001
	T10	a = 0.37906 k1 = 0.59846 n = 0.10972 k2 = 0.03657 c = 0.04926	0.9993	0.1 × 10^−3^	0.001	0.001	T11	a = 0.36001 k1 = 0.63674 n = 0.10570 k2 = 0.04163 c = 0.04620	0.9992	0.6 × 10^−3^	0.002	0.001
	T14	a = 0.35869 k1 = 0.57897 n = 0.10046 k2 = 0.03576 c = 0.04953	0.9993	0.001	0.001	0.001	T15	a = 0.32219 k1 = 0.62580 n = 0.09291 k2 = 0.04063 c = 0.04584	0.9993	0.3 × 10^−3^	0.001	0.001

	70 °C						Combined Temperatures					
Midilli-Kuck	N/O	Constants & Coffi.	R^2^	X^2^	RMSE	RSS	N/O	Constants & Coffi.	R^2^	X^2^	RMSE	RSS

	T4	a = 0.28793 k = 0.83935 b = 0.90020 n = 0.04136	0.9997	0.3 × 10^−3^	0.1 × 10^−3^	0.1 × 10^−5^	T5	a = 0.32395 k = 0.72077 b = 0.67054 n = 0.04234	0.9997	0.1 × 10^−3^	0.2 × 10^−3^	0.1 × 10^−5^
	T8	a = 0.27778 k = 0.92688 b = 0.86071 n = 0.04077	0.9997	0.001	0.9 × 10^−3^	0.1 × 10^−3^	T9	a = 0.31449 k = 0.81319 b = 0.94544 n = 0.04138	0.9997	0.5 × 10^−5^	0.3 × 10^−3^	0.1 × 10^−5^
	T12	a = 0.29737 k = 0.75329 b = 0.52704 n = 0.04554	0.9998	0.001	0.001	0.1 × 10^−3^	T13	a = 0.31405 k = 0.69600 b = 0.63143 n = 0.04681	0.9998	0.5 × 10^−5^	0.3 × 10^−3^	0.5 × 10^−5^
T16	a = 0.23942 k = 0.79481 b = 0.69655 n = 0.03836	0.998	0.1 × 10^−4^	0.001	0.001	T17	a = 0.30077 k = 0.71593 b = 0.61675 n = 0.04570	0.9998	0.3 × 10^−3^	0.3 × 10^−3^	0.5 × 10^−5^

Hii Model	T4	a = 0.29641 k1 = 0.66088 n = 0.09424 k2 = 0.04017 c = 0.04990	0.9996	0.5 × 10^−3^	0.002	0.2 × 10^−3^	T5	a = 0.33642 k1 = 0.60710 n = 0.10734 k2 = 0.04016 c = 0.04623	0.9996	0.6 × 10^−3^	0.002	0.001
	T8	a = 0.27218 k1 = 0.89665 n = 0.08512 k2 = 0.03971 c = 0.05235	0.9992	0.001	0.002	0.001	T9	a = 0.26258 k1 = 0.60394 n = 0.10204 k2 = 0.03957 c = 0.04627	0.9992	0.001	0.002	0.9 × 10^−3^
	T12	a = 0.29610 k1 = 0.68013 n = 0.09593 k2 = 0.04483 c = 0.05894	0.9995	0.9 × 10^−3^	0.5 × 10^−3^	0.0024	T13	a = 0.31860 k1 = 0.60507 n = 0.13036 k2 = 0.46305 c = 0.05390	0.9994	0.001	0.002	0.001
	T16	a = 0.25556 k1 = 0.67633 n = 0.09564 k2 = 0.49711 c = 0.04579	0.9993	0.001	0.002	0.8 × 10^−3^	T17	a = 0.30660 k1 = 0.61192 n = 0.12564 k2 = 0.48971 c = 0.05396	0.9992	0.8 × 10^−3^	0.002	0.001

Drying Constants and Coefficients (Const. & Coffic.); T2–T5 (OGS); T6–T9 (SGS); T10–T13 (US 28 kHz); T14–T17 (US 40 kHz) drying at all temperatures; Coefficient of Determination (R^2^); Chi-Square (X^2^); Root Mean Squared Error (RMSE); Residual Sum of Squares (RSS).

Among the applied models, Midilli-Kuck present the best fitting model on our data, showed that the, R^2^ 0.9995 to 0.9998, X^2^ 0.001 to 0.5 × 10^−5^, RMSE 0.001to 0.1 × 10^−3^, and RSS ≤ 0.001 to 0.1 × 10^−5^, closely followed by Hii model, with the R^2^ 0.9992 to 0.9996, X^2^ 0.001 to 0.1 × 10^−3^, RMSE 0.001 0.3 × 10^−3^, and RSS 0.001 to 0.8 × 10^−3^ (Table 2). While other model data was Page R^2^ ≥ 0.9991, Logarithmic model R^2^ ≥ 0.9989, Newton model R^2^ ≥ 0.9985, Two term exponential model R^2^ ≥ 0.9981, Henderson & Pabis Model R^2^ ≥ 0.9977….. at all drying temperatures, respectively. However, multiple investigations found that R^2^ is not the sole statistical metric for selecting and evaluating nonlinear mathematical models [49,50]. The high R^2^ and low X^2^ values of these models indicated strong fit between experimental and predicted data, indicating that all residuals are near to zero or randomly distributed [51]. Thus, X^2^, RMSE, and RSS were also considered.

**Fig. 2:**
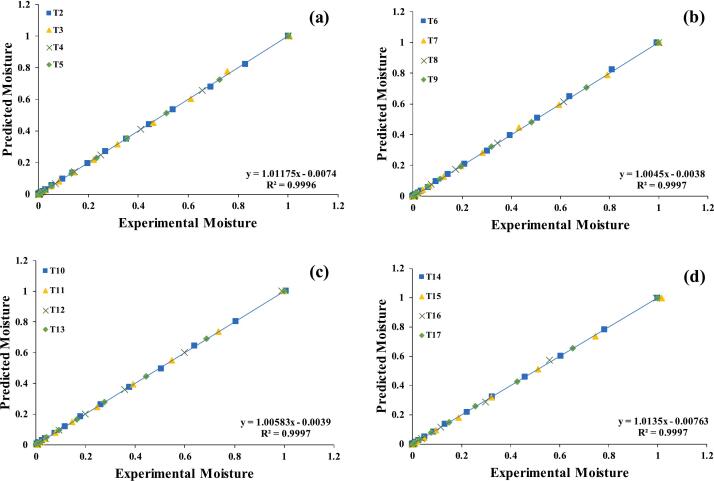
a–d: Validation of the Midilli-Kuck model by comparing quinoa predicted vs. experiment data. OGS 50 °C (T2); 60 °C (T3); 70 °C(T4); C.T (T5) (a); SGS 50 °C (T6); 60 °C (T7); 70 °C(T8); C.T (T9) (b), US 28 kHz 50 °C (T10); 60 °C (T11); 70 °C(T12); C.T (T13) (c), US 40 kHz 50 °C (T14); 60 °C (T15); 70 °C(T16); C.T (T17) (d).

**Fig. 3:**
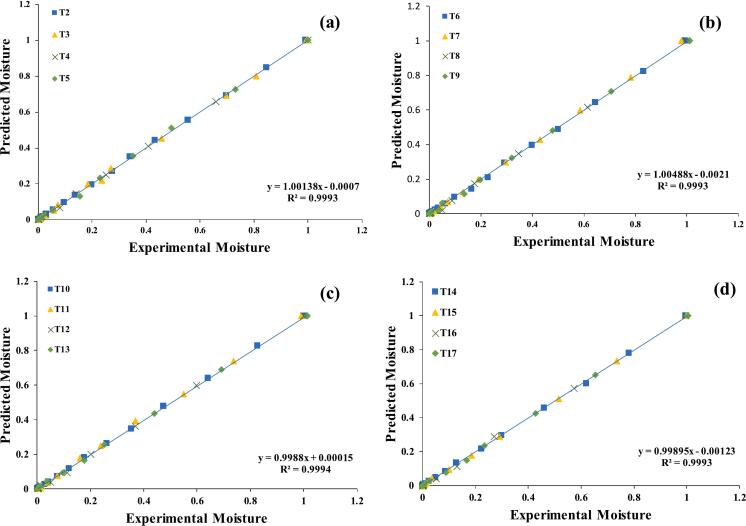
a–d: VALIDATION of the Hii model by comparing quinoa predicted vs. experiment data. OGS 50 °C (T2); 60 °C (T3); 70 °C(T4); C.T (T5) (a); SGS 50 °C (T6); 60 °C (T7); 70 °C(T8); C.T (T9) (b), US 28 kHz 50 °C (T10); 60 °C (T11); 70 °C(T12); C.T (T13) (c), US 40 kHz 50 °C (T14); 60 °C (T15); 70 °C(T16); C.T (T17) (d).

According to the observations, the Midilli-Kuck model fit well pre-treated assisted germinated quinoa grains dried in a hot air dryer at all drying temperatures presenting highest R^2^ & lowest X^2^, RMSE, RSS, followed by Hii model. Several scientific studies have found the Midilli-Kuck model, excellent fit for various agricultural products, including buckwheat (R^2^ ≥ 0.9991) [52], Ginger (R^2^ ≥ 0.9994) [53], soybean (R^2^ ≥ 0.9991) [54] and Opuntia seeds (R^2^ ≥ 0.999) [55]. The Hii model was the second best-fitting model in HAD after the Midilli-Kuck model. Rashid et al. [46], reported that the Hii model effectively described the drying kinetics of black rice, with high (R^2^ ≥ 0.997), low RMSE (≤0.001), and RSS (≤0.00). Similarly, T.Q & Jittanit [56], found the Hii model suitable for jasmine brown rice, sweet potato [22] and for pumpkin seeds [57], with R^2^ ≥ 0.999 at temperatures (50–80 °C). Fig. 2a–d and Fig. 3a–d show a comparison between the observed moisture ratio and the predicted moisture ratio obtained from the Midilli-Kuck and the Hii model for pre-treated assisted germinated quinoa grains that were dried at all temperatures. The results showed that the expected and actual moisture ratio values were very close to each other. Data from the statistical study and the coefficient of determination can be found in Table 2. It was found that as the drying temperature increase, the drying rate constant (k) number increase too. This means that as the temperature increase, the drying curve got steeper, which means that the material dried faster.

#### 3.1.3 Thermodynamic functions

Table 3 shows the thermodynamic functions (entropy, Gibbs, and enthalpy) and the minimum energy needed to extract the specified amount of water. Our results showed that higher drying temperatures resulted in lower enthalpy (ΔH), indicating less energy required for drying. Enthalpy decreased as temperature increased from 50 °C to 70 °C, with values of 15.496 kJ/mol to 15.329 kJ/mol for OGS, 19.341 kJ/mol to 18.834 kJ/mol for SGS, 25.723 kJ/mol to 25.557 kJ/mol for US 28 kHz and 15.570 kJ/mol to 15.486 kJ/mol for US 40 kHz, respectively. Araújo et al. [58], found that the product behaves as surface diffusivity increases owing to water vapour partial pressure, but air partial pressure remains constant. Ghibate et al. [59] observed similar behaviour, demonstrated at high temperature resulted lower ΔH in pomegranate peels and seeds. Wanderley et al. [60], found a positive ΔH (11.12 kJ/mol) in drying pomegranate peels, indicating an endothermic reaction.

**Table 3:** Effect of pre-treatments assisted germination on Thermodynamics functions.

Treatments	D.T	ΔH (kJ/mol)	ΔG(kJ/mol)	ΔS (kJ/mol)	Ec (kWh)
OGS	50 °C	15.496	46.308	−15.352	11.8301
	60 °C	15.412	48.107	−15.268	9.8814
	70 °C	15.329	50.127	−15.183	9.2461
	C.T	15.412	48.348	−15.267	9.7298

SGS	50 °C	19.341	46.410	−18.856	11.2625
	60 °C	18.917	48.190	−18.772	9.2149
	70 °C	18.834	50.458	−18.687	8.8512
	C.T	18.917	48.362	−18.772	9.3212

US 28 kHz	50 °C	25.723	45.799	−25.582	10.2625
	60 °C	25.640	48.155	−25.496	9.2807
	70 °C	25.557	50.216	−25.411	9.5183
	C.T	25.640	48.095	−25.496	9.7068

US 40 kHz	50 °C	15.570	46.534	−15.426	10.8049
	60 °C	15.486	48.343	−15.341	9.8512
	70 °C	15.403	50.374	−15.256	8.6080
	C.T	15.486	48.462	−15.341	9.6519

Drying temperatures (D.T); Only germinated seeds (OGS); Soaked germinated seeds (SGS); Ultrasound 28 kHz (US 28 kHz); Ultrasound 40 kHz (US 40 kHz); Combined temperatures (C.T); enthalpy (ΔH); Gibbs free energy (ΔG); entropy (ΔS); Energy consumption (Ec).

The positive Gibbs free energy increase with temperature indicated non-spontaneous drying under these study conditions. Among pre-treatments, 70 °C dried samples had the greatest ΔG values (50.12–50.48 kJ/mole), whereas lower temperatures had the lowest values (45.79–46.53 kJ/mole) (Table 3). A positive Gibbs free energy suggests an endergonic process that needed environmental energy [61]. Numerous studies on agricultural products' thermodynamics show that drying results for pomegranate seeds and peels match those of Santos et al. [62], who observed an increase in ΔG for acuri slices at 60–90 °C. According to the present studies, desorption is non-spontaneous, hence positive results are anticipated. The Gibbs free energy increased with temperature, suggesting non-spontaneous. Drying reduces moisture by transferring energy from the environment [63].

A decrease in entropy (ΔS) with increasing temperature indicates a better order in the system [58]. As drying temperature increased, ΔS dropped. Similar to enthalpy, ΔS decreased as temperature increase (Table 3). Da Silva et al. [64], found that entropy ΔS decreases with increasing temperature, indicating a greater degree of order at lower temperatures due to less water molecule excitation. Negative entropy values can be attributed to structural alterations in the adsorbent. Increasing drying air temperature and product water vapour partial pressure stimulate water molecules and reduce viscosity. These factors collectively enhance the rate of water diffusion and decrease entropy in the process [65]. In this study, the modulus entropy of the sonicated samples decreased, which are in line with other recent published studies [66,67].

Table 3 shows the *Ec* for the pre-treatments assisted germinated quinoa grains dried at various temperatures. Overall, OGS shows the highest *Ec* across all conditions, with values ranging from 9.2461 kWh to 11.8301 kWh. In contrast, SGS has slightly lower *Ec*, ranging from 8.8512 kWh to 11.2625 kWh. The US 28 & US 40 ultrasonic treatments generally consume less energy, with US 40 being the most efficient at 8.608 kWh at 70 °C and US 28 being slightly higher. *Ec* generally decreases with increasing temperature [68]. High air temperatures led to release of moisture by create more water vapour pressure within the kernels [69]. However, this increase in energy usage also produces a higher amount of water vapor during the drying process, which is due to the prolonged drying time needed at lower temperatures [70]. Similar outcomes have been observed for the air ultrasonic drying of corn husks & rice when the energy required for drying air exceeds the energy saved due to the reduction in drying time [71].

### 3.2 Techno functional properties

Quinoa exhibited superior functional properties and thus can be proposed for incorporation in conventionally consumed and novel functional foods. Data presented in Table 4 revealed that thermal processing brought significant changes in the functional properties of quinoa, such as Flour dispersablity (FD %), bulk density tapped (BDT), bulk density loose (BDL), Swelling power (SP), Foam Capacity (FC), Foam Stability (FS), True/Particle Density (TPD). FD is an index of its rehydration or reconstitute ability in water and reveals the hydrophobic action by determining the tendency of flour to move away from water molecules [72]. The control sample showed the highest value at 92.06 %, while the lowest values were observed at US 40 kHz at 50 °C (81.11 %) & SGS at 70 °C (82.97 %) (Table 4). This suggests that higher thermal and ultrasonic treatments lead to a structural modification, reducing the ability to retain flour dispensability percentage. Our data are in agreement with Sharma et al [38], who found in pre-treatment assisted germination decrease the FD % from 91.25 % in control to 73.33 % in pre-treatment assisted germinated quinoa grain (p ≤ 0.05). Sahni and Sharma [73], observed similar trend for thermally processed alfalfa and attributed it to protein denaturation caused by reduction in its solubility. Similar results have been found by Vela et al. [15], demonstrating that the FD was lowered in Ultrasound processing as compare to other treatments. In our study the FD values range from 81.11 to 92.06 % of raw and pre-treatments assisted germinated quinoa samples; demonstrating that the flour will easily reconstitute to fine consistent pudding or dough during mixing [74].

**Table 4:** Techno functional properties of pre-treated assisted germinated quinoa grains.

Treatments	D.T	FD %	BDT(g/cm^3^)	BDL(g/cm^3^)	SP (g/g)	FC %	FS %	TPD g/cm^3^
Control		92.06 ± 0.45a	0.38 ± 0.01a	0.54 ± 0.00a	2.98 ± 0.02g	19.53 ± 0.07j	51.54 ± 0.59m	1.22 ± 0.04d

OGS	50 °C	87.44 ± 0.44d	0.34 ± 0.01bcd	0.48 ± 0.01b	3.4 ± 0.04cd	22.3 ± 0.29e	58.61 ± 0.91i	1.21 ± 0.00d
	60 °C	87.49 ± 0.55d	0.31 ± 0.04ef	0.45 ± 0.00de	3.46 ± 0.04cd	22.72 ± 0.27de	66.39 ± 0.71g	1.29 ± 0.01c
	70 °C	82.77 ± 0.33gh	0.32 ± 0.00ef	0.47 ± 0.00c	2.61 ± 0.04h	20.37 ± 0.02i	52.96 ± 0.57l	1.36 ± 0.02abc
	C.T	86.53 ± 0.27de	0.27 ± 0.01gh	0.46 ± 0.00cd	2.95 ± 0.08g	21.61 ± 0.39fg	69.04 ± 0.32f	1.32 ± 0.03bc

SGS	50 °C	89.08 ± 0.51c	0.34 ± 0.02bc	0.44 ± 0.01e	3.34 ± 0.07de	21.29 ± 0.35gh	70.36 ± 0.14e	1.37 ± 0.04abc
	60 °C	85.17 ± 0.49ef	0.25 ± 0.00i	0.41 ± 0.00g	3.21 ± 0.08ef	23.69 ± 0.29a	68.57 ± 0.47f	1.34 ± 0.03bc
	70 °C	82.97 ± 0.12gh	0.32 ± 0.00def	0.48 ± 0.00b	2.31 ± 0.03i	21.6 ± 0.20fg	54.31 ± 0.18k	1.38 ± 0.02ab
	C.T	83.79 ± 0.45fgh	0.27 ± 0.01gh	0.45 ± 0.01e	3.12 ± 0.05f	22.58 ± 0.11de	56.47 ± 0.45j	1.37 ± 0.04abc

US 28 kHz	50 °C	91.33 ± 0.56ab	0.38 ± 0.00a	0.46 ± 0.01c	3.79 ± 0.02b	23.48 ± 0.25b	58.17 ± 0.31i	1.31 ± 0.02bc
	60 °C	89.08 ± 0.54c	0.29 ± 0.00g	0.39 ± 0.00h	3.94 ± 0.03a	23.15 ± 0.12cd	72.14 ± 0.53d	1.33 ± 0.02bc
	70 °C	82.37 ± 0.71hi	0.33 ± 0.00cde	0.36 ± 0.01i	3.52 ± 0.05c	20.9 ± 0.06hi	62.58 ± 0.96h	1.38 ± 0.03ab
	C.T	82.23 ± 0.73hi	0.36 ± 0.00b	0.43 ± 0.00f	3.48 ± 0.03cd	21.42 ± 0.32gh	65.16 ± 0.53g	1.38 ± 0.01ab

US 40 kHz	50 °C	81.11 ± 0.81i	0.28 ± 0.01gh	0.36 ± 0.00i	3.87 ± 0.07b	22.14 ± 0.23ef	78.02 ± 0.31c	1.44 ± 0.02a
	60 °C	84.06 ± 0.90fg	0.27 ± 0.00hi	0.36 ± 0.00i	3.86 ± 0.07b	23.24 ± 0.23bc	81.76 ± 0.58a	1.33 ± 0.00bc
	70 °C	86.49 ± 0.41de	0.36 ± 0.00b	0.42 ± 0.01f	2.48 ± 0.07h	20.59 ± 0.25i	72.64 ± 0.62d	1.31 ± 0.02bc
	C.T	89.78 ± 0.46c	0.31 ± 0.00f	0.35 ± 0.00i	3.75 ± 0.02b	21.21 ± 0.22gh	79.78 ± 0.14b	1.34 ± 0.03bc

Data are pretested in means ± standard deviation of three replicates (n = 3); Drying temperatures (D.T); Only germinated seeds (OGS); Soaked germinated seeds (SGS); Ultrasound 28 kHz (US 28 kHz); Ultrasound 40 kHz (US 40 kHz); Combined temperatures (C.T); Flour dispersablity (FD); Bulk density tapped (BDT); Bulk density loose (BD); Swelling Power (SP); Foam Stability (FS); Foam Capacity (FC); True/Particle Density (TPD); (a-m); letters indicate statistical differences by Tukey’s test (p ≤ 0.05).

Furthermore Table 4 shows that, control sample exhibited the highest values for BDT, BDL, while the lowest values were found in SGS at 60 °C followed by US 40 at 60 °C, respectively (p ≤ 0.05). These values suggests that higher processing temperatures and ultrasonic treatments cause structural expansion, reducing the compactness of the particles. The small drop in flour's bulk density might be because complex substances like starch and proteins break down because of the change that occurs during germination [75]. Because of this, the low mass density would be beneficial when making complementary foods products [76].

The SP results show that temperature and treatment type significantly impact water absorption. The control sample had 2.98 g/g, while OGS and SGS treatments increased at 50 to 60 °C (3.4 to 3.46 g/g, 3.34 to3.21 g/g) but dropped sharply at 70 °C (2.61 g/g, 2.31 g/g) due to structural breakdown. US 28 kHz enhanced SP at 60 °C (3.94 g/g) while US 40 kHz (3.87 to 3.86 g/g), but dropped at 70 °C (2.48 g/g) due to excessive heat (p ≤ 0.05). In line with the results of the flours under study, it has been generally shown that US treatments result in higher SP values, as measured in samples from millet [77], oats [78] & barley [79]. Together, these findings point to a consistent sonication impact that is unaffected by the quinoa grains [15]. An increase in SP might result from sonication's impact on the amorphous portion of starch granules, which facilitates better water absorption and more swelling than in native flours [80].

The foam capacity of SGS and US 40 kHz-treated samples at 60 °C exhibited the highest values, measuring 23.69 % and 23.15 %, respectively. Similarly, US 28 kHz treatment at 60 °C yielded a foam capacity of 23.485 %. However, increasing the temperature to 70 °C resulted in a significant reduction in foam formation, with OGS (20.37 %), SGS (21.6 %), US 28 kHz (20.9 %), and US 40 kHz (20.59 %) showing decreased values. This reduction can be attributed to heat-induced protein denaturation (p ≤ 0.05). Furthermore, the highest foam stability was observed in US 40 kHz-treated samples at 60 °C, with a value of 81.76 %. In contrast, the lowest foam stability was recorded in OGS at 70 °C (52.96 %). Notably, all treated samples exhibited higher foam stability compared to the control (51.54 %), indicating that ultrasonic treatments enhance foam retention. The true particle density values ranged from 1.21 to 1.44 g/cm^3^, with minimal fluctuations observed in the germinated samples. Our results are consistent with Vela et al. [15] and Ray et al. [81] demonstrated that, FC & FS% of quinoa improved sonication as compared to pre-treatments. Higher foaming capacity of the fractions makes it useful to improve the leavening and textural properties in cake and confectionery products.

Table 5 presents the gelation behaviour of different treatment groups at varying solution concentrations & the gelation status was assessed across different solution concentrations (2 %, 4 %, 6 %, 8 %, 10 %, 12 %, 14 %, 16 %, 18 %, 20 %, and 22 %) and classified as “No gel,” “Partial gel,” or “Strong gel”. At lower concentrations (2 %–8%), no gelation was observed in any treatment group. The onset of gelation typically occurred at 12 %, where “Partial gel” formation began. In control sample no gels was formed up to 12 %, while in pre-germination treatments groups, most samples exhibited “Partial gel,” with some groups, such as OGS, SGS, and ultrasound-treated samples (US28 kHz and US40 kHz). At lower concnetration 16 % strong gels were found in all treatments groups only dried at lower temperatures, while higher concentrations (20 %–22 %), all treatments resulted in strong gels formation at all drying temperatures, indicating that an increase in solution concentration promotes gelation.

**Table 5:** Least gelation capacity of pre-treated assisted germinated quinoa grains.

Treatments	D.T	Solution%										
		2	4	6	8	10	12	14	16	18	20	22
Control												

OGS	50 °C											
	60 °C											
	70 °C											
	C.T											

SGS	50 °C											
	60 °C											
	70 °C											
	C.T											

US 28 kHz	50 °C											
	60 °C											
	70 °C											
	C.T											

US 40 kHz	50 °C											
	60 °C											
	70 °C											
	C.T											

Drying temperatures (D.T); Only germinated seeds (OGS); Soaked germinated seeds (SGS); Ultrasound 28 kHz (US 28 kHz); Ultrasound 40 kHz (US 40 kHz); Combined temperatures (C.T);  No gel;  Partial gel;  Strong gel.

Among the treatments, ultrasound-treated samples (US28 and US40) showed faster gelation at intermediate concentrations compared to other groups, suggesting that ultrasound processing enhances gelation. In food systems, ultrasonic technology is crucial and may be a useful substitute technique for enhancing food's gelling qualities [82]. Our data are in agreement with Ray et al. [81], least gelation capacity of the quinoa samples ranged between 12 & 18 %. The author further found no gel was found in control sample at 12 %, and begin to strong gel at 20 %. Samples with high starch and protein content illustrate that there is a physical competition for water between starch gelatinisation and proteins gelation which influences the gelling capacity [83].

**Fig. 4:**
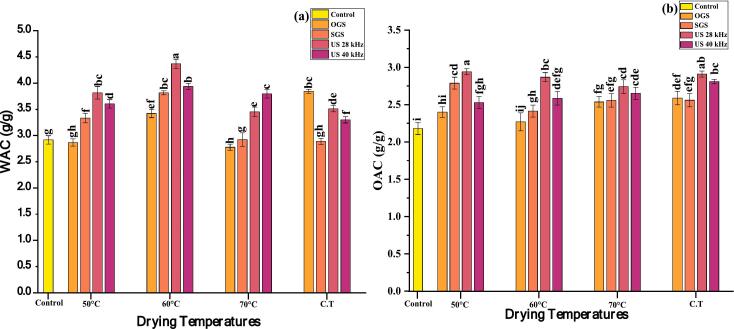
(a-b): Effects of pre-treatments on germinated quinoa grains followed by dried in hot air dryer at various temperatures. Only germinated seeds (OGS); soaked germinated seeds (SGS); Ultrasound 28 kHz (US 28 kHz); Ultrasound 40 kHz (US 40 kHz); Combined temperatures (C.T); Water absorption capacity (WAC) (a); Oil absorption capacity (OAC) (b). Data were taken in replicate, (a–i): letters show statistical differences by Tukey’s test (p ≤ 0.05).

Water absorption capacity (WAC) reflects the ability of the sample to retain water, which is influenced by structural properties and pre-treatments germinated conditions. Fig. 4a shows that control sample had a WAC of 2.92 g/g, pre-treatment assisted germination led to significant variations. Overall, the WAC of OGS was ranged from 2.77 to 3.84 g/g, SGS 2.88 to 3.81 g/g, US 28 3.50 to 4.36 g/g and US 40 3.33 to 3.93 g/g (p ≤ 0.05). Among the tested condition the US 28 exhibited an increasing trend 60 °C, 4.36 g/g followed by US 40 at 60 °C (3.93 g/g), indicating enhanced WAC due to heat induced structural modifications that increase hydrophilic interactions. Overall, WAC increased with moderate temperature and ultrasonic exposure, highlighting enhanced WAC due to modifications in the sample matrix. The WAC largely impact texture, taste and appearance of food; thus, can act as strong indicators of quality measures in food manufacturing. Our results are in line with, Ray et al. [76], who found that WAC of varied quinoa samples ranged between 1.93 and 3.24 g/g. Earlier researchers showed values of WHC in the range 2.31–2.56 g/g for the quinoa fractions, while the whole quinoa had the value of 2.29 g/g [84]. In addition, Donmez et al. [85], also suggest that the WAC of the food components are mainly influenced by their chemical composition. High WAC allows bakers to add higher amount of water during preparation; thus, improves dough quality and handling leading to extended freshness with increased yield.

Oil absorption capacity (OAC) is an important property for formulation of emulsions needed in several food products, the principle of physical entrapment of oil in an emulsion play a key role in the product taste and texture. OAC determines the ability of a material to of flour is crucial for functional gel, thereby influencing the texture, stability, and mouthfeel of the gel [86]. Fig. 4b shows that control sample had an OAC of 2.18 g/g, with variations observed across treatments. All pre-treatments assisted germination showed increasing OAC values with temperature, Overall the WAC of OGS was ranged from 2.42 to 2.58 g/g, SGS 2.41 to 2.78 g/g, US 28 2.74 to 2.94 g/g and US 40 2.52 to 2.86 g/g, reaching the highest increase at 2.94 g/g, particularly at US 28 at 50 °C temperatures (2.94 g/g), followed by US 28 at 60 °C 2.86 g/g (p ≤ 0.05); indicating that ultrasonic cavitation creates microstructural modifications that increase oil retention. Overall, OAC improved with both thermal and ultrasonic treatments, suggesting enhanced oil-binding properties due to structural modifications and increased surface area. Our results are in line with Olawuni et al. [87], demonstrated quinoa grains OAC range from 2.19 to 3.10 g/g. While Ray et al. [81] found slightly lower than our results 1.15 g/g to 2.11 g/g. Difference in the absorption capacities (water and oil) of quinoa fractions are mainly attributed to the protein structures present in the ingredients.

### 3.3 Minerals profile

The mineral composition of raw and pre-treatments assisted germinated quinoa grains is shown in Table 6. Minerals are essential in human health for maintaining and promoting an overall mental and physical wellness in terms of taking integral part in the development and maintenance of body tissues, blood muscles, nerve cells, bones, and teeth [88]. Quinoa is a significant source mineral, appropriate for a balanced diet like calcium, magnesium, and potassium [89]. The table presents the mineral content (Mg, Ca, Fe, Cu, K, Mn & Zn) of quinoa grains subjected to various pre-treatments, drying temperatures, and ultrasonic frequencies. The results indicate that ultrasonic treatments, particularly at 28 kHz, significantly increase the mineral content, especially Mg, Ca, K, and Mn. For example, the US 28 kHz treatment at 50 °C shows the highest Mg (142.71 ± 1.42 mg/100 g), Ca (86.3 ± 3.47 mg/100 g), and K (446.15 ± 7.04 mg/100 g) levels, compared to the control group, which exhibits lower mineral concentrations. Similarly, US 40 kHz at 50 °C achieves the highest Fe content (5.64 ± 0.20 mg/100 g) while at 60°C the highest content of K were found (448.71 ± 10.19 mg/100 g) than control. Cu & Zn contents also show significant increases, with US 28 kHz at 50 °C having the highest Cu (1.03 ± 0.04 mg/100 g) and US 28 kHz at 60 °C showing the highest Zn (3.65 ± 0.10 mg/100 g). The levels of Mn remain relatively stable across treatments, with a peak observed in the OGS treatment at 60 °C. Overall, ultrasonic treatments at 28 kHz enhance the mineral content, particularly at lower drying temperatures (50 °C), suggesting that such pre-treatment methods may improve the nutritional value of quinoa grains. Ultrasonic pre-treatment was found to improve the Mg, Ca, Fe, K & Zn, in quinoa grains. This is likely attributed to the increased cellular permeability induced by ultrasound, which promotes the release of bound minerals from the seed’s matrix, making them more available for absorption. The higher concentrations of these minerals in the ultrasonic-treated groups suggest that ultrasound may play a role in improving mineral retention during the drying process. Our findings are consistent with Maldonado-Alvarado et al. [90], reported Zn content in germinated quinoa grains, ranging from 1.73 ± 0.25 to 3.83 ± 0.08 mg/100 g. However, the author observed slightly lower Ca (64.88 ± 0.84 to 76.76 ± 4.87 mg/100 g) and Fe (4.68 ± 0.48 to 4.81 ± 0.25 mg/100 g) content. Cañarejo-Antamba et al. [91], reported comparable Mg (97.66 ± 2.52 to 152.33 ± 4.04 mg/100 g) and Fe (3.85 ± 0.05 to 3.97 ± 0.11 mg/100 g) content, but lower Ca content (14.66 ± 1.52 to 60.33 ± 3.21 mg/100 g). In agreement with our results, Bhinder et al. [88], observed similar values for Ca (50.76 ± 2.51 to 89.75 ± 3.23 mg/100 g), Cu (0.84 ± 0.02 to 1.54 ± 0.09 mg/100 g), and Mn (2.56 ± 0.08 to 3.39 ± 0.11 mg/100 g) content. However, the author reported higher Fe content (10.96 ± 0.14 to 12.84 ± 0.2 mg/100 g). These variations can be attributed to differences in region, growing conditions, and variety [92]. Additionally, other studies [8,88] reported similar K (402.94 ± 6.56 to 562.15 ± 21.77 mg/100 g) and Mn (2.56 ± 0.08 to 3.39 ± 0.11 mg/100 g) content. Ultrasound-assisted germination have significantly increase the content Mg, Ca, & K possibly due to ultrasound pre-treatments improve seed permeability, activating enzymes that break down stored compounds, mobilizing nutrients, and improving the overall germination process [93]. These effects work together to enhance the availability of minerals during and after the germination process.

**Table 6:** Minerals profile of pre-treated assisted germinated quinoa grains mg/100 g.

Treatments	D.T	Mg	Ca	Fe	Cu	K	Mn	Zn
Control		122.42 ± 2.22g	71.12 ± 6.26b	4.48 ± 0.24b	1.25 ± 0.17a	425.04 ± 3.56b	3.53 ± 0.25a	3.43 ± 0.17ab

OGS	50 °C	136.29 ± 4.98abcd	83.64 ± 2.83ab	5.59 ± 0.34a	0.81 ± 0.04cd	437.79 ± 5.40ab	3.23 ± 0.17bc	2.53 ± 0.25cd
	60 °C	139.5 ± 2.71abc	84.66 ± 3.50ab	5.06 ± 0.24ab	0.93 ± 0.10bcd	435.84 ± 4.60ab	3.36 ± 0.03abc	2.61 ± 0.31cd
	70 °C	125.75 ± 4.22efg	78.94 ± 8.36ab	5.11 ± 0.41ab	0.91 ± 0.04bcd	436.2 ± 3.64ab	3.27 ± 0.08abc	2.94 ± 0.03bcd
	C.T	127.55 ± 4.31defg	82.56 ± 3.37ab	5.52 ± 0.44ab	0.82 ± 0.02cd	432.7 ± 4.99ab	3.25 ± 0.06abc	2.34 ± 0.09d

SGS	50 °C	131.4 ± 2.50bcdefg	85.24 ± 3.97a	5.21 ± 0.36ab	0.85 ± 0.04bcd	440.5 ± 4.35ab	3.5 ± 0.04ab	3.15 ± 0.04abc
	60 °C	135.12 ± 2.38abcde	81.58 ± 4.86ab	5.39 ± 0.26ab	0.87 ± 0.03bcd	436.6 ± 4.10ab	3.33 ± 0.03abc	3.29 ± 0.14ab
	70 °C	128.77 ± 2.40defg	83.37 ± 5.02ab	5.21 ± 0.31ab	0.95 ± 0.03bcd	432.56 ± 5.36ab	3.24 ± 0.06bc	2.6 ± 0.31cd
	C.T	136.26 ± 4.22abcd	82.04 ± 3.41ab	5.11 ± 0.25ab	0.95 ± 0.03bc	441.15 ± 10.52ab	3.3 ± 0.07abc	2.62 ± 0.25cd

US 28 kHz	50 °C	142.71 ± 1.42a	86.3 ± 3.47a	5.55 ± 0.15a	1.03 ± 0.0434b	446.15 ± 7.04a	3.5 ± 0.07ab	3.38 ± 0.06ab
	60 °C	128.77 ± 2.40ab	83.37 ± 5.02a	5.23 ± 0.42ab	0.78 ± 0.01cd	437.07 ± 13.13ab	3.25 ± 0.05abc	3.65 ± 0.10cd
	70 °C	125.03 ± 3.32fg	77.49 ± 5.29ab	5.08 ± 0.59ab	0.76 ± 0.01d	430.31 ± 6.08ab	3.28 ± 0.06abc	2.58 ± 0.35cd
	C.T	133.4 ± 2.22abcdef	83.4 ± 5.48ab	5.22 ± 0.48ab	0.95 ± 0.02bc	433.06 ± 6.00ab	3.11 ± 0.04c	2.82 ± 0.17bcd

US 40 kHz	50 °C	135.92 ± 5.67abcd	85.64 ± 3.10a	5.64 ± 0.20a	0.93 ± 0.05bcd	440.01 ± 8.76ab	3.54 ± 0.11a	3.25 ± 0.21ab
	60 °C	139.55 ± 2.88abc	84.61 ± 4.17ab	5.44 ± 0.18ab	0.94 ± 0.04bcd	448.71 ± 10.19a	3.51 ± 0.05ab	3.65 ± 0.23a
	70 °C	130.33 ± 3.01cdefg	78.87 ± 5.55ab	5.45 ± 0.39ab	0.86 ± 0.09bcd	432.66 ± 5.54ab	3.3 ± 0.07abc	3.36 ± 0.29ab
	C.T	132.28 ± 3.54bcdef	76.61 ± 4.45ab	5.19 ± 0.23ab	0.77 ± 0.02cd	441.04 ± 9.07ab	3.23 ± 0.03bc	2.61 ± 0.20cd

Data are pretested in means ± standard deviation; Data of first three replicates (n = 3); Drying temperatures (D.T); Only germinated seeds (OGS); Soaked germinated seeds (SGS); Ultrasound 28 kHz (US 28 kHz); Ultrasound 40 kHz (US 40 kHz); Combined temperatures (C.T); Magnesium (Mg); Calcium (Ca); Iron (Fe) Copper (Cu); Potassium (K); Manganese (Mn); Zinc (Zn); (a–l), letters shows statistical differences by Tukey’s test (p ≤ 0.05).

### 3.4 Phenolic acid profile

**Fig. 5:**
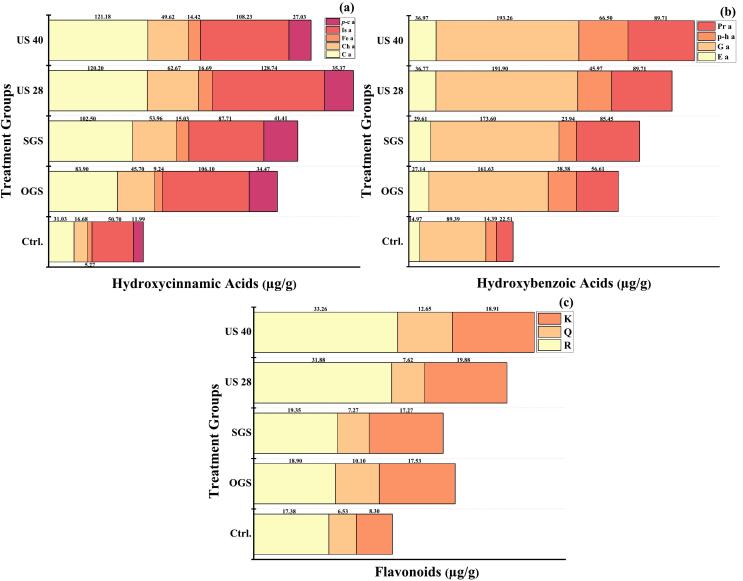
Grouped bar chart; Effects of pre-treatments on germinated quinoa grains followed by dried in hot air dryer at various temperatures. Only germinated seeds (OGS); Soaked germinated seeds (SGS); Ultrasound 28 kHz for 30 min (US 28); Ultrasound 40 kHz for 30 min (US 40); Caffeic acid (Ca a); Chlorogenic acid (Ch a); Ferulic acid (Fe a); Isoferulic acid (Is a); *p*-coumaric acid (*P*-c a); Ellagic acid (E a); p-Hydroxybenzoic acid (p-h a); protocatechuic (pr a); Rutin (R); Quercetin (Q) & Kampferol (K). Data were taken in replicate (p ≤ 0.05).

Quinoa's bioactive compounds mostly detected in the outer layers, serve as a chemical defence against insects and pathogens [89]. Research has shown that the high concentration of bioactive components in quinoa seeds is linked to their antioxidant activity [94]. Table 7 shows the impact of different pre-treatment-assisted germination methods and drying temperatures on the hydroxycinnamic and benzoic acid content of quinoa grains. US 28 showed the highest concentrations of caffeic acid (139.91 µg/g at 60 °C) and chlorogenic acid (68.79 µg/g at 50 °C), ferulic acid (22.58 µg/g at 50 °C), while SGS produced the highest levels of isoferulic acid (156.55 µg/g at 50 °C) closely followed by US 28 (143.73 µg/g at 50 °C) and ferulic acid (16.45 µg/g at 50 °C). The highest *p*-coumaric acid concentration (57.77 µg/g) was observed in US 40 at 60 °C (p ≤ 0.05). Overall US 40 showed moderate results, with its highest caffeic acid at 60 °C (134.82 µg/g), ferulic acid at 60 °C (59.25 µg/g) & isoferulic at 60 °C (125.23 µg/g) (Fig. 5a). Our results fall in line with ferulic acid 23.7 to 150 and *p*-coumaric acid 17.1 to 80.0 µg/g in quinoa grains while undetected sinipic acids [95,96]. In addition, Złotek et al. [97], reported *p*-coumaric 37.25 to 58.41 in quinoa. In hydorxybenzoic acid US 40 at 60 °C exhibited the highest concentration at 48.02 µg/g ellagic acid, followed by SGS at 60 °C (31.37 µg/g) and OGS at 50 °C (29.74 µg/g), while US 40 at 50 °C (85.12 µg/g) showed the highest concentration Protocatechuic acid, followed by OGS at 50 °C (65 µg/g). In terms of Gallic acid a significant increases were observed with US 40 at 50 °C (218.18 µg/g), US 28 at 60 °C (214.85 µg/g) & OGS at 60 °C (183.26 µg/g), indicating the substantial enhancement of this compound under these conditions. US 40 found 85.12 µg/g p-Hydroxybenzoic acid, while lowest were found in SGS 14.08 µg/g (Fig. 5b). Our study results are in agreement with previous findings who found Gallic acid 4.78 to 295.11 µg/g, Protocatechic acid 8.02 to 126.27 µg/g, p-Hydroxybenzoic acid 22.13 to 54.57 µg/g, Chlorogenic acid 7.86 µg/g, Caffeic acid 4.81 µg/g, *p*-coumaric acid 15.92 to 48.39 µg/g, Ferulic acid 72.62 to 118.18 µg/g ellagic acid 12.03–14.29 µg/g in germinated and un-germinated quinoa grain [[97], [98], [99]]. Gallic acid was the major HBA detected in free form and ranged from 218.18 ± 1.68 to 89.39 ± 1.40 µg/g, higher in US 40 dried at 50 °C among all treated samples. Furthermore, Qian et al. [100], also reported a higher content of Gallic acid the most prevalent phenolic acid in quinoa grains. Overall, these results indicate that treatments, particularly US 40 at 50 °C, significantly enhance the concentrations of key phenolic acids. We found 2.5–4 fold increase in phenolic acid profile of pre-treatments assisted germinated quinoa grains. Our results are consistent with previous findings of Zhang et al. [101] found 3-fold increase in phenolic acid content of buckwheat after germination. While Vicente-Sánchez et al. [102] reported 4-fold in germinated quinoa grains. Furthermore, we found that higher temperatures at 70 °C generally reduced the concentrations of these compounds, indicating that moderate temperatures are more effective for preserving phenolic acids. Similar results has been reported by Ramos-Pacheco et al. [8], reported a decrease in phenolic acid content of germinated quinoa grains dried at 70 °C. Overall, ultrasound-assisted germination, particularly at 28 kHz, proved to be the most efficient method for increasing hydroxycinnamic acid content in quinoa grains, with the 60 °C drying condition yielding the best results. SGS also demonstrated strong performance across multiple hydroxycinnamic acids, making it a viable alternative. The duration and conditions of germination play a significant role in these enhancements [9]. Ultrasound pre-treatment facilitates better water absorption and accelerates metabolic processes by creating micro-bubbles in the liquid medium [103]. These micro-bubbles collapse during the process, generating localized heat and shockwaves that can break down cell walls, disrupt seed coat integrity, and enhance nutrient absorption, leading to improved germination rates [104]. This mechanical stress also increases the activity of enzymes that are involved in metabolic pathways during germination, promoting faster seedling growth. These processes help in maximizing germination efficiency and optimizing the production of valuable bioactive compounds in the early stages of plant development.

**Table 7:** Phenolic acids profile of pre-treated assisted germinated quinoa grains µg/g.

Treatments	D.T	Ca-a	Ch-a	Fe-a	Is-a	*P*-c-a	El-a	Ga-a	P-H-a	Pr-a
Control		31.03 ± 0.12g	26.68 ± 0.15f	5.27 ± 0.93e	50.7 ± 1.59hi	11.99 ± 0.67c	14.97 ± 0.37b	89.39 ± 1.40i	14.39 ± 0.25d	32.51 ± 1.67fg

OGS	50 °C	90.07 ± 1.33e	44.51 ± 2.35bcde	8.05 ± 0.26e	85.85 ± 1.44fgh	31.91 ± 0.88b	29.74 ± 1.43ab	158.26 ± 1.08gh	65 ± 0.24abcd	109.76 ± 1.27abc
	60 °C	105.12 ± 2.46bcde	63.24 ± 1.65bc	14.34 ± 1.04cd	137.53 ± 0.35bcd	35.29 ± 0.33b	27.14 ± 0.68ab	183.26 ± 2.27e	23.42 ± 1.38cd	49.54 ± 0.31defg
	70 °C	51.32 ± 2.24f	27.77 ± 2.53ef	6.35 ± 1.39e	73.23 ± 0.242ghi	30.15 ± 0.83b	25.06 ± 1.34ab	151.62 ± 1.50h	32.36 ± 1.90abcd	21.98 ± 2.87fg
	C.T	89.11 ± 1.81e	21.05 ± 1.04ef	8.24 ± 1.39e	127.82 ± 0.66cde	40.56 ± 0.84ab	26.64 ± 0.71ab	153.4 ± 0.65gh	32.75 ± 1.61abcd	35.16 ± 0.96efg

SGS	50 °C	98.29 ± 0.81cde	58.45 ± 1.32bc	16.45 ± 0.12bcd	143.73 ± 1.96bc	40.13 ± 2.83ab	30.32 ± 0.90ab	184.84 ± 1.11e	48.76 ± 0.32d	52.66 ± 0.93fefg
	60 °C	131.87 ± 0.41ab	72.23 ± 0.73ab	18.05 ± 0.70bc	156.55 ± 1.43a	28.85 ± 1.72b	31.37 ± 1.54ab	194.51 ± 0.77d	14.08 ± 0.53d	108.8 ± 1.67abc
	70°C	87.25 ± 0.92e	45.68 ± 0.98bcde	13.16 ± 1.30d	40.74 ± 2.32i	33.44 ± 1.02b	26.25 ± 0.85ab	153.9 ± 0.64gh	15.09 ± 0.79abcd	91.4 ± 0.42bcde
	C.T	91.45 ± 1.10e	39.49 ± 2.14cde	12.49 ± 0.44d	80.68 ± 1.87fghi	34.32 ± 1.13b	30.5 ± 1.87ab	161.15 ± 0.38gh	17.83 ± 1.17d	88.96 ± 1.73bcde

US 28 kHz	50 °C	129.21 ± 1.99abc	68.79 ± 0.83a	22.58 ± 2.11a	135.31 ± 1.06bcdef	40.9 ± 2.99ab	38.44 ± 2.69ab	197.29 ± 1.60cd	54.34 ± 0.75abcd	42.08 ± 2.13defg
	60°C	139.91 ± 1.91a	61.77 ± 0.716ab	18.14 ± 1.59b	152.75 ± 1.55ab	37.72 ± 0.93ab	37.6 ± 1.08ab	214.85 ± 0.45bc	55.77 ± 0.74abcd	113.47 ± 2.80ab
	70 °C	88.94 ± 0.41e	52.26 ± 0.87bcd	12.85 ± 0.11d	88.9 ± 2.28efgh	30.43 ± 1.81b	37.39 ± 2.99ab	183.14 ± 0.47e	26.61 ± 0.33bcd	81.29 ± 2.47cdef
	C.T	123.95 ± 0.26abcd	58.94 ± 0.10bc	13.2 ± 0.30d	104.47 ± 0.65cdefg	32.43 ± 2.02b	33.66 ± 0.04ab	163.64 ± 0.35fg	47.16 ± 2.68abcd	80.47 ± 0.48cdef

US 40 kHz	50 °C	128.85 ± 2.77abc	53.11 ± 0.38bcd	15.79 ± 0.40bcd	94.16 ± 1.78defgh	26.09 ± 1.06bc	38.44 ± 1.47ab	218.18 ± 1.68a	85.12 ± 0.14a	89.84 ± 1.10bcde
	60°C	134.82 ± 0.71ab	59.25 ± 2.84bc	14.74 ± 0.45bcd	125.23 ± 1.21cdef	57.77 ± 1.88a	48.02 ± 1.33a	205.97 ± 1.27cd	74.21 ± 0.47abc	119.68 ± 1.71a
	70 °C	96.28 ± 1.96de	39.32 ± 1.24cde	13.32 ± 2.23d	102.72 ± 0.45cdefg	25.69 ± 0.75bc	26.47 ± 1.28ab	172.5 ± 1.39f	27.21 ± 0.62bcd	103.66 ± 1.70abcd
	C.T	124.77 ± 0.60abcd	47.01 ± 0.65bcde	13.83 ± 0.09d	110.82 ± 0.74cdefg	27.51 ± 0.14bc	34.95 ± 3.63ab	185.08 ± 0.76e	79.46 ± 0.00ab	105.66 ± 1.64abcd

Data are pretested in means ± standard deviation of three replicates (n = 3); Data are pretested in means ± standard deviation; Data of first three replicates (n = 3); Drying temperatures (D.T); Only germinated seeds (OGS); Soaked germinated seeds (SGS); Ultrasound 28 kHz (US 28 kHz); Ultrasound 40 kHz (US 40 kHz); Combined temperatures (C.T); Caffeic acid (Ca-a); Chlorogenic acid (Ch-a); Ferulic acid (Fe-a); Isoferulic acid (Is-a); *P*-coumaric acid (*P*-c-a); Ellagic acid (El-a); Gallic acid (Ga-a); P-Hydroxybenzoic acid (P-H-a); Protocatechuic acid (Pr-a); expressed as µg/g; (a–i); letters show statistical differences by Tukeys test (p ≤ 0.05).

### 3.5 Flavonoids profile

Table 8 indicate that the US 40 treatment at 60 °C exhibited the highest levels of rutin 22.62 ± 0.56 µg/g, quercetin 17.58 ± 1.84 µg/g & kaempferol 22.49 ± 0.35 µg/g among the different pre-treatment conditions. This suggests that US40 treatment at 60 °C could be the most effective for enhancing the bioactive compounds in quinoa grains. The US28 treatment also performed well, especially at 50 °C, with rutin and kaempferol levels of 21.25 ± 0.06 µg/g and 22.07 ± 0.46 µg/g, respectively, indicating that this treatment is also beneficial for increasing these compounds (Fig. 5c). In contrast, SGS treatments at 50 °C showed significant increases in rutin (21.22 ± 1.75 µg/g) and kaempferol (21.85 ± 0.82 µg/g), though quercetin levels were relatively lower compared to the US40 and US28 treatments (p ≤ 0.05). The substantial increase in Kaempferol content in US 28 and 40 at 50 °C indicates that ultrasound treatment plays a crucial role in upregulating flavonoid synthesis, supporting previous findings [105]. According to earlier research, after 72 h of germination, the levels of rutin, quercetin increases 3–4 folds in buckwheat and quinoa grains [88,101] (p ≤ 0.05). Our data are in agreement with previous findings who demonstrated quinoa grains rutin 7.64 to 52.14, quercetin 6.51 to 17.61 µg/g, Kaempferol 5.28 to 21.52 µg/g [98,99]. While Bhinder et al. [88] found an increase in germinated quinoa grains Rutin from 10.38to 13.43 µg/g & quercetin 5.6 to 10.53 µg/g. Furthermore, Yang et al. [3] reported in quinoa grains Rutin 14.97 to 30.96, quercetin 18.55 to 37.43 ± 0.1 and kampferol 10.07 to 18.61 µg/g (p ≤ 0.05). Interestingly, higher temperatures 70 °C generally resulted in lower levels of Flavonoids, particularly quercetin, which had the lowest values at 70 °C for SGS and US28 treatments [11]. This suggests that higher temperatures may not be optimal for preserving or increasing the levels of flavonoids.

**Table 8:** Flavonoids profile of pre-treated assisted germinated quinoa grains µg/g.

Treatments	D.T	Flavonoids profile µg/g		
		Rutin	Quercetin	Kaempferol
Control		12.38 ± 1.07g	6.35 ± 0.82ef	8.3 ± 0.24g

OGS	50 °C	16.27 ± 0.16e	16.43 ± 0.29ab	19.29 ± 0.63c
	60 °C	16.39 ± 0.32e	12 ± 0.263c	17.06 ± 0.97cd
	70 °C	12.31 ± 0.18g	5.61 ± 0.31f	15.88 ± 0.87de
	C.T	15.65 ± 1.47ef	7.37 ± 1.55def	17.91 ± 0.50cd

SGS	50 °C	21.22 ± 1.75b	9.38 ± 2.47cde	21.85 ± 0.82b
	60 °C	11.68 ± 0.87g	6.08 ± 1.97ef	17.39 ± 1.12cd
	70 °C	10.86 ± 1.12h	3.76 ± 2.86f	13.59 ± 0.25ef
	C.T	15.22 ± 0.90f	9.87 ± 0.91cde	15.58 ± 0.71de

US 28 kHz	50 °C	21.25 ± 0.06b	7.88 ± 1.67def	22.07 ± 0.46b
	60 °C	19.65 ± 0.22c	11.24 ± 0.87cd	18.95 ± 0.37c
	70 °C	17.61 ± 5.06d	3.57 ± 0.29f	17.18 ± 0.97cd
	C.T	21.01 ± 1.29b	7.79 ± 1.68def	17.9 ± 0.06cd

US 40 kHz	50 °C	21.57 ± 0.87b	12.15 ± 1.91c	24.51 ± 0.17a
	60 °C	22.62 ± 0.56a	17.58 ± 1.84a	22.49 ± 0.35b
	70 °C	18.03 ± 0.79d	7.74 ± 1.29def	12.7 ± 1.25f
	C.T	20.22 ± 1.13c	13.15 ± 1.01bc	15.96 ± 0.64de

Data are pretested in means ± standard deviation of three replicates (n = 3); Data are pretested in means ± standard deviation; Data of first three replicates (n = 3); Drying temperatures (D.T); Only germinated seeds (OGS); Soaked germinated seeds (SGS); Ultrasound 28 kHz (US 28 kHz); Ultrasound 40 kHz (US 40 kHz); Combined temperatures (C.T); expressed as µg/g; (a–g) letters show statistical differences by Tukey’s test (p ≤ 0.05).

Overall, US 40 at 60 °C emerged as the most effective treatment, demonstrating the highest concentrations of Rutin and Quercetin, while US 40 50 °C was the most effective for Kaempferol (Table 8). These findings suggest that specific treatments, particularly US 40, significantly influence flavonoid than the results of the germination treatment, probably because ultrasonic makes the seed shell and accelerates the hydration process [106], leading to changes in the molecular structure and catalysis of enzymes, triggering the defence reaction systems, and enhancing the production of secondary metabolites such as the polyphenols [107]. The cavitation and mechanical effects of ultrasonic enhance the permeability of cell membranes and promote diffusion and transmembrane transport of ions and metabolites [108]. According to Wang’s report [13], the flavonoid content of mung bean seeds was significantly increased after ultrasonic treatment and germination for 48 h and was significantly higher than that of the samples germinated alone, which is consistent with the results of this study.

### 3.6 Crystalline property XRD

**Fig. 6:**
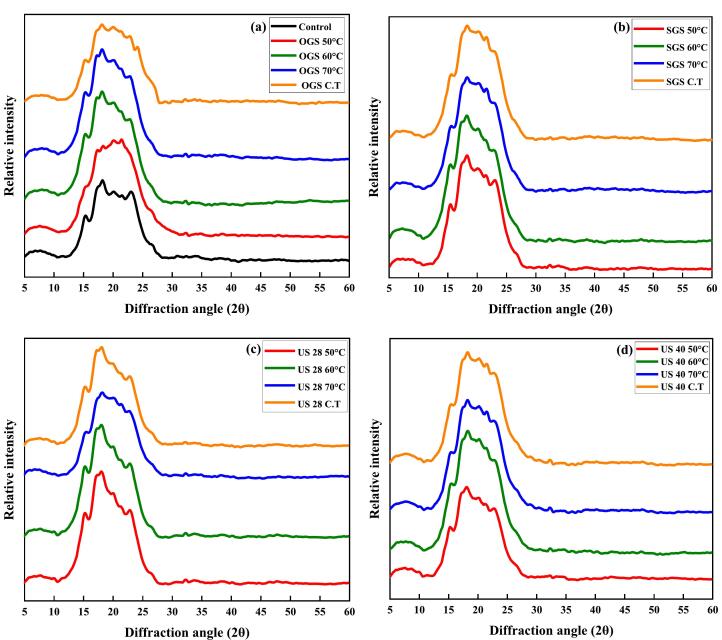
a–d; X-ray diffraction pattern and relative crystallinity of pre-treatments assisted germinated quinoa grain dried at all temperatures. OGS (a); SGS (b); US 28 kHz (c); US 40 kHz (d).

Fig. 6a-d depicts XRD patterns revealing distinct structural alterations with diffraction peaks observed at 15° to 23° (2θ), due to the pretreatments assisted germination of quinoa grains followed by dried at various temperatures.. All investigated conditions exhibit a typical A-type crystalline structure. Changes in the overall intensities were noted in the germinated samples, however there were no significant shifts in the prominent peaks in the XRD patterns in the present study. Changes in quinoa grain crystallinity are linked to variations in the intensities of pre-treated samples. Several authors have previously reported on the reduction in crystallinity in germinated grain [109,110]. Germination results in improved crystallinity, with 60 °C exhibiting the strong peaks among all tested conditions. In contrast, increased temperature treatments, particularly at 70 °C, tend to reduce peak intensity, The OGS and SGS treatments show a significant enhancement in crystallinity Fig. 6a-b, while ultrasound-assisted treatment at a higher frequency enhances crystallinity more effectively than SGS and OGS treatments alone Fig. 6c-d. Overall, all treatments result in a decrease in crystallinity with increasing temperature, with the most significant structural changes occurring in the C.T. condition. Crystalline structure is typical of the starch granules seen germinated samples. A similar outcome was observed in germinating mung beans [111] and sorghum [112]. According to previous findings diffraction peaks between 14°, 15°, 16°, 18°, 21°, and 24° (2θ) can be seen in the diffractogram of quinoa by Ramos-Pacheco et al. [8], rice [113].

### 3.7 SEM

**Fig. 7:**
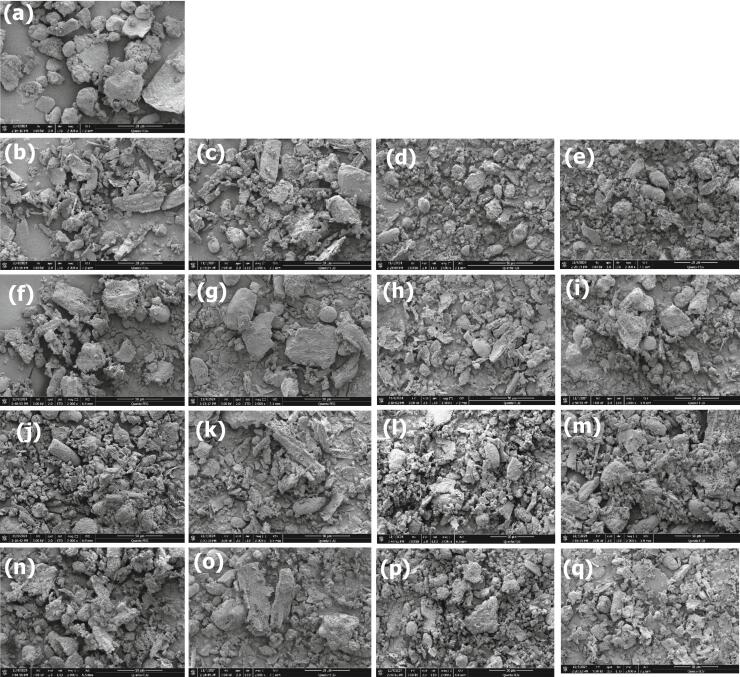
a-q: scanning electron microscope images of the quinoa samples (a–q) magnification 2000× with scale bar (50 µm). Control (a); OGS (b–e), SGS (f–i); US 28 kHz (j–m); US 40 kHz (n–q), germinated samples dried at all drying temperatures.

Control and pre-treated germinated samples, scanning electron micrographs, 2000x with a scale bar of 50 µm shown in (Fig. 7a–q). The SEM revealed aggregations of germinated quinoa subjected to several temperatures. A film-like material seems to cover and connect the aggregates possibly a protein matrix with bonded to the lipids & starch clumps [114].

Control sample (Fig. 7a) showed noticeable differences with respect to pre-treated sample. The control image (a) displays larger, with smooth, rounded surfaces, serving as a baseline for comparison. In contrast, OGS images (b–e) reveal progressive fragmentation with increasing temperatures. At 50 °C (b), surface appear eroded and fragmented with a visible micro cracks and rougher surfaces compared to the control, while 60 °C (c) shows increased disintegration. Further fragmentation occurs at 70 °C (d), and C.T (e) leads to partial aggregation. SGS (f–i) exhibit similar trends, with increased disintegration at higher temperatures. At 50 °C (f), particles appear slightly larger and more cohesive, while 60 °C (g) shows increased fragmentation. Higher temperatures (h) result in highly disrupted particles. US 28 (j–m) Particles are significantly eroded, especially at 60 °C (k). Surface pitting and debris accumulation are evident at 50 °C (j), with increased breakage and fragmentation at 60 °C (k). C.T images (m) leads to mixed morphology. US at 40 kHz (n–q) yielding smaller, uniform particles. Furthermore, US treatments, are the most effective in breaking down particles, while OGS & SGS in moderate fragmentation. Our findings provide further evidence that the starch aggregates are bound to the lipids in addition to being embedded in a protein matrix. A comparable result has been found by Khan et al. [21] in quinoa Ye et al. [115] in rice and Annor et al. [116] in millet.

## 4 Conclusion

Pre-treatments assisted germination can be an effective technique for enhancing the quality profile of quinoa grains. But the extent of improvements depend on the pre-germination treatments and drying temperature. The first creative justification of this study was determining the optimal model for the drying kinetics of quinoa grains dried in HAD. The next step was to determine how the quality profile was impacted by various pre-treatments assisted germination, and drying temperatures. For the first time this study underscores the ultrasounds assisted germinated quinoa grain in a control germinated chamber offers deeper understanding as compared to other methods. US 40 kHz at low temperature exhibited superior functional properties, for example “Strong gel” formation observed at 16 -18 % solution concentration, while the control sample showed no gel until 20 %. Ultrasonic treatment at 28 kHz and 60 °C resulted in the highest WAC, while the highest OAC was achieved at 50 °C. Regarding mineral content, ultrasonication at 28 kHz yielded the highest levels of Mg, Ca, Fe, and K in quinoa. Furthermore, US 40 kHz treatment resulted in the highest content of Ca, Fe, Mn, and Zn. US 28 kHz significantly enhanced the concentrations of hydroxycinnamic, with the highest levels of caffeic, chlorogenic & ferulic acid. In terms of hydroxybenzoic acids, the highest concentration was recorded in US 40 kHz followed by US 28 kHz. Overall 2.5 to 4-fold increase in flavonoids & phenolic acid profiles was observed; Indicating that the combination of frequency and temperature is the most effective for enhancing the quinoa quality profile, making it the best method compared to other treatments in the study. In contrast, higher temperature 70 °C drying led to a reduction in overall quinoa quality profile. Conducting such studies would provide strong evidence for the possible use of germinated quinoa flours as a functional component in creating novel meals or dietary supplements. Our study suggests that germination processes favour the accumulation of phenolic compounds in the grains. The results encourage the application of germinated quinoa as potential ingredients for the development of innovative and nutritious products.

## Funding sources

We acknowledge the financial support from 10.13039/501100001809National Natural Science Foundation of China (32472381, 32172259), the 10.13039/100006190Key Research and Development Project of Henan Province (231111111800) and Henan Provincial Natural Science Foundation (242300420465).

## CRediT authorship contribution statement

**Jabir Khan:** Writing – review & editing, Writing – original draft, Project administration, Investigation, Formal analysis, Data curation, Conceptualization. **Yang Li:** Writing – review & editing, Methodology. **Palwasha Gul:** Writing – review & editing, Visualization, Validation, Software. **Qingyun Li:** Writing – review & editing, Visualization. **Kunlun Liu:** Visualization, Validation, Supervision, Project administration, Methodology.

## Declaration of competing interest

The authors declare that they have no known competing financial interests or personal relationships that could have appeared to influence the work reported in this paper.
